# Mechanism of action of adapalene for treating EGFR‐TKI‐induced skin disorder

**DOI:** 10.1111/1759-7714.15249

**Published:** 2024-02-20

**Authors:** Chihiro Mimura, Tatsuya Nagano, Nanako Miwa, Kanoko Matsumura, Jun Yamada, Hiroki Satoh, Ratoe Suraya, Daisuke Hazama, Daisuke Tamura, Masatsugu Yamamoto, Motoko Tachihara, Yoshihiro Nishimura, Kazuyuki Kobayashi

**Affiliations:** ^1^ Division of Respiratory Medicine Kobe University Graduate School of Medicine Kobe Japan; ^2^ Department of Respiratory Medicine Kobe City Nishi‐Kobe Medical Center Kobe Japan; ^3^ Department of Respiratory Medicine Takatsuki General Hospital Takatsuki Japan; ^4^ Tamura Clinic Takarazuka Japan; ^5^ Department of Respiratory Medicine Kitaharima Medical Center Ono Japan

**Keywords:** acneiform eruption, afatinib, erlotinib, molecular‐targeting agent, skin disorder

## Abstract

**Background:**

Skin disorders are the most common side effect associated with epidermal growth factor receptor‐tyrosine kinase inhibitor (EGFR‐TKI) therapy. It is important to manage skin lesions. Adapalene has been used to treat skin lesions caused by EGFR‐TKIs in some cases. The aim of this study was to investigate the functional mechanism of adapalene in erlotinib‐induced skin disorder.

**Methods:**

To analyze the effect of adapalene on skin rash, afatinib and adapalene were administered to mice. The relationship between the concentration of adapalene and skin disorders was also examined by analyzing AQP3 expression. A skin lesion model was experimentally established in human skin keratinocytes (HaCaT) by using erlotinib with TNF‐*α* and IL‐1β. We used qRT–PCR to analyze chemokine‐induced inflammation and western blotting to analyze the effects of adapalene on the NF‐κB signaling pathway. Antimicrobial peptides and adhesion factors were also examined using qRT–PCR.

**Results:**

Mice administered 0.01% adapalene had less skin inflammation than mice treated with afatinib alone. The expression level of AQP3 decreased in an adapalene concentration‐dependent manner. The mRNA levels of proinflammatory cytokines such as CCL2 and CCL27 in HaCaT cells were significantly reduced by adapalene. The expression of an antimicrobial peptide, hBD3, was upregulated after adapalene treatment. Adhesion factors, such as E‐cadherin, were significantly downregulated by EGFR‐TKI and significantly upregulated by adapalene treatment. Western blot analysis suggested that erlotinib‐induced phosphorylation of p65 was decreased by adapalene.

**Conclusion:**

We suggest that adapalene may be a possible treatment option for skin disorders induced by EGFR‐TKIs.

## Graphical abstract

This figure shows molecular mechanism of adapalene. Adapalene decreased the expression level of AQP3, proinflammatory cytokines such as CCL2 and CCL27*.* Adapalene upregulated the expression of an antimicrobial peptide, hBD3, and adhesion factors, E‐cadherin.

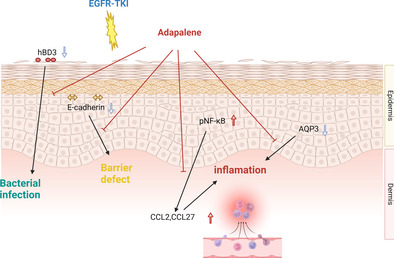

## INTRODUCTION

Lung cancer is the most common cause of cancer‐related death. In non‐small cell lung cancer (NSCLC), epidermal growth factor receptor‐tyrosine kinase inhibitors (EGFR‐TKIs) have been effective in treating *EGFR*‐mutated lung cancer.^1^, ^2^ However, the adverse events caused by EGFR‐TKI therapy are unique from those caused by conventional cytotoxic chemotherapy. Among these adverse events, skin disorders occur frequently reduce patient quality of life.^3^ It is also known that the severity of EGFR‐TKI‐induced skin rashes are dose‐dependent, with increased drug exposure leading to increased incidence and severity.^4^ It is important to control skin disorders since they can hinder further EGFR‐TKI treatment.

EGFR‐TKI‐induced skin disorders include papulopustular rush, hair loss, eye/eyelash abnormalities, periungual/nail alterations and xerosis/pruritus.^5^ In particular, maculopapular rash has been reported to be present in 50%–100% of patients.^6^ Important skin tissues such as epidermal tissue, hair follicles, and tissues surrounding the nail express EGFR, and EGFR‐TKI treatment inhibits the activity of EGFR in the skin, resulting in the abnormal keratinization of keratinocytes. In addition, chemokines such as C‐C motif chemokine 2 (CCL2), CCL5, and C‐X‐C motif chemokine ligand 10 (CXCL10) are produced by epithelial cells, causing chemokine‐induced inflammation and various skin disorders. In addition, secondary infections and barrier defects can exacerbate skin disorders.^7^ Inhibition of epidermal defense factors, such as antimicrobial peptides and intercellular adhesion factors, by EGFR‐TKIs causes epidermal infection.^8^ Specifically, some of these factors include hBD3, an antimicrobial peptide, and E‐cadherin, an adhesion factor.

The skin regulates water permeability through aquaporins (AQPs). In particular, aquaporin 3 is abundant in the epidermis, the outermost layer of the skin. Defects in AQP are known to reduce water content and delay wounding in the skin as a result of reduced water transport in keratinocytes and impaired keratinocyte migration.^9^, ^10^ Previously, it has been reported that retinoic acid can upregulate the expression of AQP3.^11^, ^12^

Adapalene is a naphthoic acid derivative similar to retinoids (vitamin A derivatives). The chemical structure of adapalene is 6‐[4‐Methoxy‐3‐(tricyclo[3.3.1.13,7]dec‐1yl)phenyl]naphthalene‐2‐carboxylic acid, and its molecular formula is C28H28O3. Its structure is highly stable, and adapalene is virtually insoluble in water, acetonitrile, or ethanol. Adapalene activates gene transcription by binding to retinoic acid receptors in the cell nucleus and exerts effects on keratinocytes in follicular epithelial cells. It is used for the treatment of acne vulgaris and is recommended for patients with acne due to skin damage caused by EGFR‐TKIs, since there have been reports that oral retinoic acid has been successful in treating skin disorders caused by EGFR‐TKI therapy.^13^ Some studies report the use of adapalene for skin damage caused by EGFR‐TKIs.^14^, ^15^ However, according to the Multinational Association for Supportive Care in Cancer (MASCC) Skin Toxicity Study Group, which is comprises international, interdisciplinary experts in dermatology, oral retinoic acid is recommended for the treatment of skin disorders caused by the use of EGFR‐TKIs.^16^ Although adapalene plays crucial roles in antiproliferative and anti‐inflammatory pathways,^17^ the therapeutic mechanism of action of adapalene in EGFR‐TKI‐induced acne‐like eczema is not clear. Therefore, the aims of this study were to analyze the effect of adapalene on EGFR‐TKI‐induced skin disorders and to clarify the mechanism of action of adapalene.

## METHODS

### Reagents

Erotinib was purchased from Chugai Pharmaceutical. Afatinib was purchased from Boehringer Ingelheim Japan. Adapalene was purchased from Tokyo Chemical Industry.

### Mice

Male Slc mice (5 weeks old) were purchased from SLC (Shizuoka, Japan). The mice were divided into five groups: control, afatinib, adapalene, afatinib and adapalene, and afatinib and low concentration 0.1% adapalene. Afatinib (0.1 mg/0.1 g) was applied to the skin of the mice. Adapalene (0.01% or 0.1%) was also administered to the mouse skin. All mice were observed for 7 days. This study was approved by the President of Kobe University after review by the Institutional Animal Care and Use Committee (permission no.: P211001) and carried out according to the Kobe University Animal Experimentation Regulations.

### Cell culture and treatment

Human skin keratinocytes (HaCat) purchased from CLS Cell Lines Service GmbH were cultured in Dulbecco's modified Eagle medium (DMEM: Wako) with 10% fetal bovine serum (FBS) and 1% antibiotic solution. All cells were grown in an incubator with 5% CO_2_ at 37°C, and the medium was replaced every 2 days. To induce inflammation, 10 ng/mL TNF‐α and 5 ng/mL IL‐1β were added for 6 h or 24 h. Erlotinib (10 μM) and various concentrations of adapalene (25 nM, 250 nM, 2000 nM) were used.

### Real‐time quantitative PCR

After treatment for 24 h, total RNA was isolated using Sepasol as previously indicated.^18^ Total RNA was reverse transcribed from RNA to cDNA using 5X Primescript RT Master Mix (Takara). Real‐time PCR was performed using TB Green Premix Ex Taq II (Takar). To stimulate cytokinesis, cells were treated with 100 ng/mL LPS. GAPDH was selected as the control. Primers were constructed for CCL2, CCL27, LL37, hBD3, and RNase‐7.

### Western blot analysis

Western blotting was performed using a previously reported protocol.^18^ In this study, the following primary antibodies were used: β‐actin (#4967), NF‐kB (#8242), and phospho‐NF‐kB (#3033). All were purchased from Cell Signaling Technology.

### Statistical analysis

Data are presented as the mean ± standard deviation (SD). Statistical analysis was used to compare differences between the two groups using Student's unpaired *t* test. A *p*‐value < 0.05 was considered statistically significant for all analyses.

## RESULTS

### Effect of adapalene on skin rash

**FIGURE 1:**
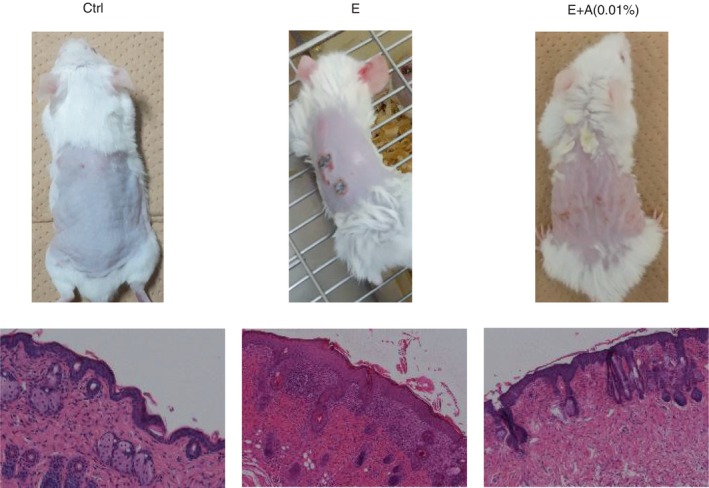
Treatment of skin damage by erlotinib and adapalene administration in mice. Mice treated with erlotinib (E) and adapalene (A) diluted to 0.01% showed less skin damage than mice treated with erlotinib alone. Ctrl, control.

Mice were observed for 7 days in the control, afatinib alone, and afatinib plus 0.01% adapalene groups, and skin rashes were observed on Day 3 in the afatinib group but not in the afatinib plus 0.01% adapalene group. Pathological findings showed less inflammation in the afatinib plus 0.01% adapalene group than in the afatinib group (Figure 1). Intriguingly, mice treated with 0.1% adapalene, with or without afatinib, showed more skin inflammation (Figure S1).

### Effect of adapalene on the expression of AQP‐3

**FIGURE 2:**
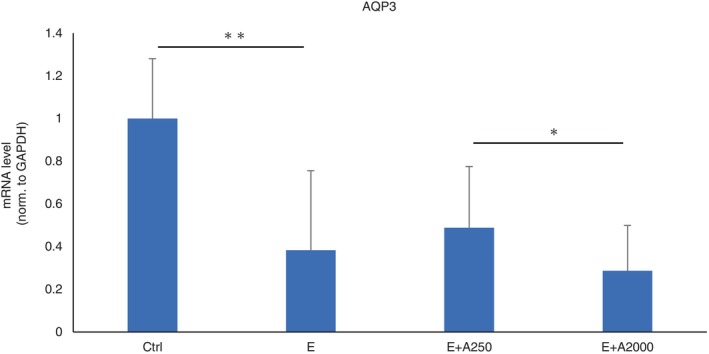
The expression of AQP3 according to the adapalene concentration. The mRNA levels of AQP3 in HaCaT cells were significantly decreased by EGFR‐TKI treatment. AQP3 expression levels decreased with increasing concentrations of adapalene. Ctrl, control. E, erlotinib 10 nM, E + A250, erlotinib 10 nM + adapalene 250 nM. E + A2000, erlotinib 10 nM + adapalene 2000 nM. Data are expressed as mean ± S.D., *p < 0.05, **p < 0.01.

To evaluate the relationship between adapalene concentrations and skin rashes, we investigated the expression of AQP3, which is present in the outer epithelial layer of the skin, by RT–PCR. HaCaT cells were treated with an EGFR‐TKI only or an EGFR‐TKI and adapalene. The results showed that the expression of AQP3 was downregulated by the EGFR‐TKI. The expression of AQP3 further decreased with higher concentrations of adapalene (Figure 2). Taken together, these results suggest that adapalene can partially ameliorate EGFR‐TKI‐induced skin disorders by downregulating AQP3.

### Role of adapalene in the inflammation of keratinocytes treated with EGFR‐TKIs

**FIGURE 3:**
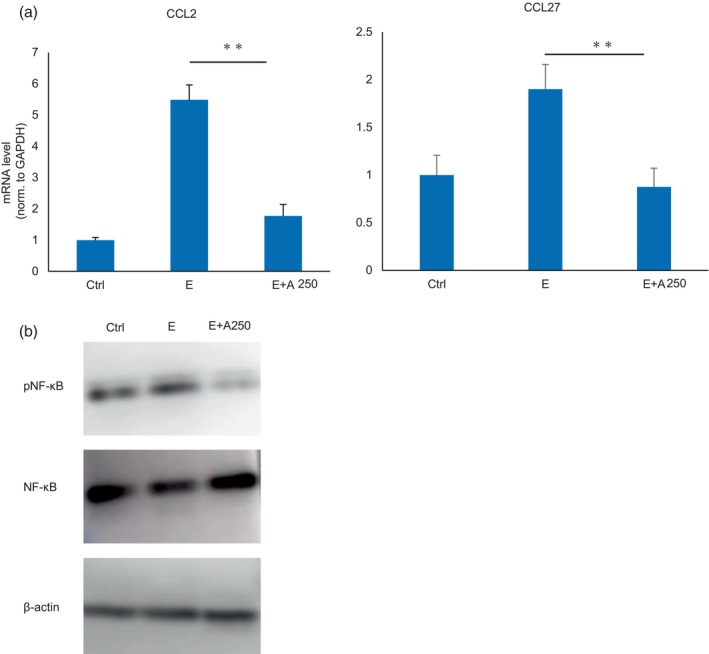
(a) Cytokine production. The mRNA levels of the proinflammatory cytokines CCL2 and CCL27 in HaCaT cells were significantly decreased by adapalene. Ctrl, control. E, erlotinib 10 nM. E + A250, erlotinib 10 nM + adapalene 250 nM. Data are expressed as mean ± S.D., **p < 0.01. (b) The effects of adapalene on the NF‐kB pathway. The effects of adapalene on the NF‐kB pathway were analyzed by western blotting. Treatment of HaCaT cells with erlotinib and adapalene downregulated the expression of pNF‐kB. Ctrl, control. E, erlotinib 10 nM. E + A250, erlotinib 10 nM + adapalene 250 nM.

The inhibitory effect of adapalene on the levels of inflammatory cytokines in the skin tissue of mice treated with EGFR‐TKIs was analyzed by RT–PCR. Human inactivated epidermal keratinocytes (HaCaT cells) were treated with an EGFR‐TKI only and an EGFR‐TKI and adapalene. The results showed that the expression of chemokines CCL2 and CCL27 was upregulated by EGFR‐TKI treatment and downregulated by adapalene treatment (Figure 3a, Figure S2).

NF‐κB, which regulates the transcription of cytokines, has been shown to be induced by EGFR‐TKI stimulation in cancer cells. We used HaCaT cells to analyze the expression of NF‐κB after EGFR‐TKI and adapalene stimulation by western blotting and elucidate the mechanism by which adapalene reduces skin inflammation. pNF‐κB was downregulated by adapalene treatment, suggesting that adapalene may ameliorate EGFR‐TKI‐induced skin damage via NF‐κB signaling (Figure 3b).

### Role of adapalene in antimicrobial peptide production

**FIGURE 4:**
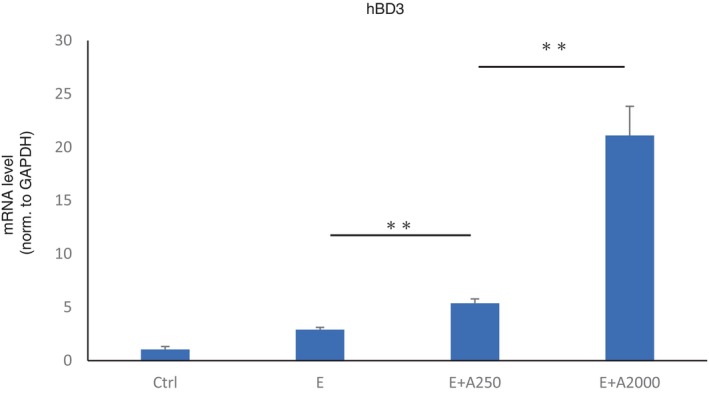
Antimicrobial peptide production. The mRNA levels of the antimicrobial peptide hBD3 in HaCaT cells were significantly increased by adapalene treatment, and the changes in hBD3 expression were dose dependent. Ctrl, control. E, erlotinib 10 nM. E + A250, erlotinib 10 nM + adapalene 250 nM. Data are expressed as mean ± S.D. **p < 0.01.

HaCat cells were used to evaluate the antimicrobial role of adapalene using RT–PCR and HaCaT cells treated with an EGFR‐TKI alone or an EGFR‐TKI with adapalene. The expression of hBD3, an antimicrobial peptide, was upregulated by adapalene treatment in a dose‐dependent manner (Figure 4). However, hBD3 was not downregulated by EGFR‐TKI treatment, contrary to our expectation.

### Role of adapalene in cell–cell adhesion

**FIGURE 5:**
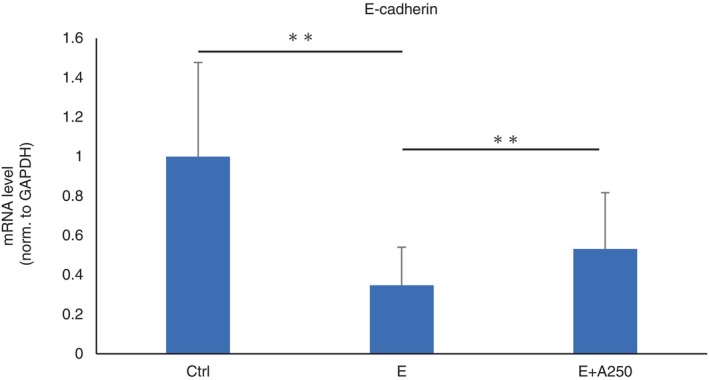
Adhesion factor production. The mRNA levels of the adhesion factor E‐cadherin in HaCaT cells were significantly decreased by EGFR‐TKI treatment and significantly increased by adapalene treatment. Ctrl, control. E, erlotinib 10 nM. E + A250, erlotinib 10 nM + adapalene 25 0 nM. Data are expressed as mean ± S.D. **p < 0.01.

The expression of E‐cadherin, an adhesion factor, was significantly downregulated by EGFR‐TKI and significantly upregulated by adapalene treatment (Figure 5).

## DISCUSSION

In this study, we investigated the effect of adapalene on EGFR‐TKI‐induced skin damage. We have experienced cases of acne‐like skin rash during treatment with EGFR‐TKI, in which the skin disorder improved with the application of adapalene.^19^ Previous reports have shown a correlation between skin rash severity and patient prognosis after treatment with erlotinib.^20^ Ameliorating skin disorders is very important for the continued administration of EGFR‐TKIs.

EGFR‐TKIs may cause skin damage by inducing aberrant keratinization and proinflammatory cytokine production in epithelial cells.^21^ Proinflammatory cytokines such as CCL2 have also been shown to be potentially involved in psoriatic skin inflammation and irritant contact dermatitis.^22^, ^23^ In this study, we demonstrated that adapalene may inhibit the production of inflammatory cytokines.

Adapalene binds to retinoic acid receptors (RAR). Adapalene treatment downregulated pNF‐κB expression, suggesting that RARs may be involved in NF‐κB expression after translocation into the nucleus. Retinoic acid has been reported to suppress NF‐κB activation,^24^ and we speculate that adapalene may exert its effects through a similar mechanism. pNF‐κB expression was downregulated, thereby reducing inflammatory cytokine expression and ameliorating the skin damage caused by EGFR‐TKI. On the other hand, although NF‐κB expression has been reported to be induced by EGFR‐TKI stimulation,^25^ no upregulation was observed in the present study.

The skin protects against infection. EGFR‐TKIs cause sterile acne, but bacterial infections, such as *Staphylococcus aureus*, may develop after prolonged periods of EGFR‐TKI treatment.^26^ Topical antimicrobial agents are an option for acne‐like skin eruptions,^27^ but long‐term use of such agents may induce bacterial resistance. Adapalene upregulates the expression of antimicrobial peptides, which may reduce the risk of bacterial infections that contribute to the skin damage caused by EGFR‐TKIs. It has also been reported that combination therapy with adapalene and clindamycin was more effective than monotherapy in treating acne vulgaris.^28^

The upregulated expression of adhesive factors with adapalene administration was demonstrated in this study. All‐trans retinoic acid has been shown to activate E‐cadherin expression via promoter hypomethylation.^29^ Previously, deletion of E‐cadherin has been shown to cause overproliferation, differentiation defects and impaired barrier formation.^30^ Its upregulated may improve skin barrier function.

Mouse experiments suggested that diluted adapalene exerts a preventive effect on skin rashes. However, high doses of adapalene have been shown to induce skin inflammation. One of the main side effects of adapalene is irritation in the early stage of topical application. A previous report also showed that some concentrations of adapalene can cause severe inflammation and exacerbate skin damage caused by EGFR‐TKIs.^31^ Previous reports have shown that in some cases, tazarotene administration was discontinued due to irritation caused by its application.^32^ Adapalene should be used once a day, but side effects may be reduced by decreasing the frequency of use or by diluting the dose.

Aquaporin expression has been implicated in the retention of epidermal water content in skin diseases.^33^ We showed that EGFR‐TKI treatment downregulated AQP3 expression and that adapalene treatment upregulated AQP3 expression. Previously, it has been reported that EGFR‐TKI decreased the expression of AQP3 and caused skin dryness in mice. One possible reason for this could be that EGFR‐TKI administration inhibits EGFR in the skin, which in turn suppresses Ras/MAPK pathway activity and AQP3 transcription.^34^ On the other hand, AQP3 expression was downregulated by high concentrations of adapalene. In a previous report, the expression of AQP3 was downregulated when retinoic acid concentrations exceeded a certain value or the exposure time was prolonged.^11^, ^35^ The causes of these changes are not clear and require further study. High concentrations of retinoic acid might contribute to skin dryness, causing changes in osmotic pressure and decreasing the expression level of AQP3.

Topical steroids are the mainstay of treatment for acneiform eruptions caused by EGFR‐TKIs, and strong topical agents are often used in Japan. Although topical steroids are an important treatment, long‐term use can lead to rosacea‐like dermatitis and skin desquamation, so concomitant use of adapalene may be expected to reduce the side effects of topical steroids. Another advantage of treatment with adapalene is that it can be used over a long period of time without adverse side effects, unlike topical steroids.

On the other hand, there are disadvantages of treatment with adapalene. Adapalene is currently recommended for the treatment of acne vulgaris only on the face, limiting its potential use. One important side effect is irritation in the early stages of use. If the skin disorder caused by EGFR‐TKI is more severe, the side effects of adapalene may be more pronounced, and it is important to treat this inflammation with a moisturizer or other combination therapy.

In this study, we were not able to determine the effect of adapalene on skin wound healing. It has been reported that retinoic acid stimulates fibroblast growth and the production of extracellular matrix by fibroblasts, although the direct effects of adapalene and dermal fibroblasts are unknown.^36^ Adapalene is a retinoid‐activated drug and may be involved in skin wound healing through similar effects. We believe this is an issue for future research.

In conclusion, adapalene suppressed inflammatory cytokine production and upregulated the expression of antimicrobial peptides and adhesion factors. An appropriate dose of adapalene may be beneficial for the treatment of skin disorders induced by EGFR‐TKIs.

## AUTHOR CONTRIBUTIONS

CM contributed to the project administration, methodology, data curation, statistical analysis, and writing the original draft. TN contributed to the conceptualization, methodology, investigation, writing and reviewing of the manuscript, and supervision. DT contributed to the conceptualization, methodology, investigation, writing and reviewing of the manuscript. NM, KM, JY, HS, and RS contributed to methodology, investigation, and writing and reviewing of the manuscript. DH, MY, MT, YN, and KK contributed to the writing and reviewing of the manuscript. All authors read and approved the final manuscript.

## CONFLICT OF INTEREST STATEMENT

The author reports no conflicts of interest in this work.

## Supporting information


**FIGURE S1.** Skin disorder of mice treated with 0.1% adapalene with or without afatinib. Both mice showed severe skin inflammation.

**FIGURE S2.** mRNA levels of indicated cytokines normalized to GAPDH.

## References

[1] Mitsudomi T , Morita S , Yatabe Y , Negoro S , Okamoto I , Tsurutani J , et al. Gefitinib versus cisplatin plus docetaxel in patients with non‐small‐cell lung cancer harbouring mutations of the epidermal growth factor receptor (WJTOG3405): an open label, randomised phase 3 trial. Lancet Oncol. 2010;11:121–128. 20022809 10.1016/S1470-2045(09)70364-X

[2] Zhou C , Wu Y‐L , Chen G , Feng J , Liu XQ , Wang C , et al. Erlotinib versus chemotherapy as first‐line treatment for patients with advanced EGFR mutation‐positive non‐small‐cell lung cancer (OPTIMAL, CTONG‐0802): a multicentre, open‐label, randomised, phase 3 study. Lancet Oncol. 2011;12:735–742. 21783417 10.1016/S1470-2045(11)70184-X

[3] Joshi SS , Ortiz S , Witherspoon JN , Rademaker A , West DP , Anderson R , et al. Effects of epidermal growth factor receptor inhibitor‐induced dermatologic toxicities on quality of life. Cancer. 2010;116:3916–3923. 20564072 10.1002/cncr.25090

[4] Yuan C , Wang B . Acneiform eruption induced by molecularly targeted agents in antineoplastic therapy: a review. J Cosmet Dermatol. 2023;22:2150–2157. 36924348 10.1111/jocd.15704

[5] Lacouture ME . Mechanisms of cutaneous toxicities to EGFR inhibitors. Nat Rev Cancer. 2006;6:803–812. 16990857 10.1038/nrc1970

[6] Chanprapaph K , Vachiramon V , Rattanakaemakorn P . Epidermal growth factor receptor inhibitors: a review of cutaneous adverse events and management. Dermatol Res Pract. 2014;2014:734249. 24723942 10.1155/2014/734249 PMC3958662

[7] Holcmann M , Sibilia M . Mechanisms underlying skin disorders induced by EGFR inhibitors. Mol Cell Oncol. 2015:e1004969. 10.1080/23723556.2015.1004969 PMC4905346 27308503

[8] Kim JM , Ji JH , Kim YS , Lee S , Oh SY , Huh SJ , et al. rhEGF treatment improves EGFR inhibitor‐induced skin barrier and immune defects. Cancers (Basel). 2020;12:3120. 10.3390/cancers12113120 33113881 PMC7692663

[9] Hara‐Chikuma M , Verkman AS . Aquaporin‐3 functions as a glycerol transporter in mammalian skin. Biol Cell. 2005;97:479–486. 15966863 10.1042/BC20040104

[10] Hara‐Chikuma M , Verkman AS . Roles of aquaporin‐3 in the epidermis. J Invest Dermatol. 2008;128:2145–2151. 18548108 10.1038/jid.2008.70

[11] Xing F , Liao W , Jiang P , Xu W , Jin X . Effect of retinoic acid on aquaporin 3 expression in keratinocytes. Genet Mol Res. 2016;15:15016951. 10.4238/gmr.15016951 26985947

[12] Bellemère G , Von Stetten O , Oddos T . Retinoic acid increases aquaporin 3 expression in normal human skin. J Invest Dermatol. 2008;128:542–548. 17943189 10.1038/sj.jid.5701047

[13] Vezzoli P , Marzano AV , Onida F , Alessi E , Galassi B , Tomirotti M , et al. Cetuximab‐induced acneiform eruption and the response to isotretinoin. Acta Derm Venereol. 2008;88:84–86. 18176767 10.2340/00015555-0330

[14] DeWitt CA , Siroy AE , Stone SP . Acneiform eruptions associated with epidermal growth factor receptor‐targeted chemotherapy. J Am Acad Dermatol. 2007;56:500–505. 17166623 10.1016/j.jaad.2006.06.046

[15] Taguchi K , Fukunaga A , Okuno T , Nishigori C . Successful treatment with adapalene of cetuximab‐induced acneiform eruptions. J Dermatol. 2012;39:792–794. 22168666 10.1111/j.1346-8138.2011.01424.x

[16] Lacouture ME , Anadkat MJ , Bensadoun R‐J , Bryce J , Chan A , Epstein JB , et al. Clinical practice guidelines for the prevention and treatment of EGFR inhibitor‐associated dermatologic toxicities. Support Care Cancer. 2011;19:1079–1095. 21630130 10.1007/s00520-011-1197-6 PMC3128700

[17] Shroot B , Michel S . Pharmacology and chemistry of adapalene. J Am Acad Dermatol. 1997;36:S96–S103. 9204085 10.1016/s0190-9622(97)70050-1

[18] Kunimasa K , Nagano T , Shimono Y , Dokuni R , Kiriu T , Tokunaga S , et al. Glucose metabolism‐targeted therapy and withaferin a are effective for epidermal growth factor receptor tyrosine kinase inhibitor‐induced drug‐tolerant persisters. Cancer Sci. 2017;108:1368–1377. 28445002 10.1111/cas.13266 PMC5497794

[19] Tachihara M , Tokunaga S , Tamura D , Kobayashi K , Funada Ya NY . Successful Treatment with adapalene for EGFR‐TKI‐induced acneiform eruptions. Jpn J Lung Cancer. 2014;54:978–982.

[20] Wacker B , Nagrani T , Weinberg J , Witt K , Clark G , Cagnoni PJ . Correlation between development of rash and efficacy in patients treated with the epidermal growth factor receptor tyrosine kinase inhibitor erlotinib in two large phase III studies. Clin Cancer Res. 2007;13:3913–3921. 17606725 10.1158/1078-0432.CCR-06-2610

[21] Holcmann M , Sibilia M . Mechanisms underlying skin disorders induced by EGFR inhibitors. Mol Cell Oncol. 2:e1004969. 10.1080/23723556.2015.1004969 PMC4905346 27308503

[22] Novoszel P , Holcmann M , Stulnig G , De Sa Fernandes C , Zyulina V , Borek I , et al. Psoriatic skin inflammation is promoted by c‐Jun/AP‐1‐dependent CCL2 and IL‐23 expression in dendritic cells. EMBO Mol Med. 2021;13:e12409. 33724710 10.15252/emmm.202012409 PMC8033525

[23] Shibuya R , Ishida Y , Hanakawa S , Kataoka TR , Takeuchi Y , Murata T , et al. CCL2–CCR2 signaling in the skin drives surfactant‐induced irritant contact dermatitis through IL‐1β–mediated neutrophil accumulation. J Invest Dermatol. 2022;142:571–582.e9. 34560074 10.1016/j.jid.2021.07.182

[24] Gu B , Miao J , Fa Y , Lu J , Zou S . Retinoic acid attenuates lipopolysaccharide‐induced inflammatory responses by suppressing TLR4/NF‐kappa B expression in rat mammary tissue. Int Immuno pharmacol. 2010;10:799–805. 10.1016/j.intimp.2010.04.022 20438866

[25] Blakely CM , Pazarentzos E , Olivas V , Asthana S , Yan JJ , Tan I , et al. NF‐κB‐activating complex engaged in response to EGFR oncogene inhibition drives tumor cell survival and residual disease in lung cancer. Cell Rep. 2015;11:98–110. 25843712 10.1016/j.celrep.2015.03.012 PMC4394036

[26] Peuvrel L , Bachmeyer C , Reguiai Z , Bachet JB , André T , Bensadoun RJ , et al. Semiology of skin toxicity associated with epidermal growth factor receptor (EGFR) inhibitors. Support Care Cancer. 2012;20:909–921. 22361824 10.1007/s00520-012-1404-0

[27] Lacouture ME , Anadkat MJ , Bensadoun R‐J , Bryce J , Chan A , Epstein JB , et al. Clinical practice guidelines for the prevention and treatment of EGFR inhibitor‐associated dermatologic toxicities. Support Care Cancer. 2011;19:1079–1095. 21630130 10.1007/s00520-011-1197-6 PMC3128700

[28] Dogra S , Sumathy TK , Nayak C , Ravichandran G , Vaidya PP , Mehta S , et al. Efficacy and safety comparison of combination of 0.04% tretinoin microspheres plus 1% clindamycin versus their monotherapy in patients with acne vulgaris: a phase 3, randomized, double‐blind study. J Dermatolog Treat. 2021;32:925–933. 32020824 10.1080/09546634.2020.1720579

[29] Woo Y‐J , Jang KL . All‐trans retinoic acid activates E‐cadherin expression via promoter hypomethylation in the human colon carcinoma HCT116 cells. Biochem Biophys Res Commun. 2012;425:944–949. 22910408 10.1016/j.bbrc.2012.08.038

[30] Arnaud T , Rodrigues‐Lima F , Viguier M , Deshayes F . Interplay between EGFR, E‐cadherin, and PTP1B in epidermal homeostasis. Tissue Barriers. 2023;11:2104085. 35875939 10.1080/21688370.2022.2104085 PMC10364651

[31] Chayahara N , Mukohara T , Tachihara M , Fujishima Y , Fukunaga A , Washio K , et al. Adapalene gel 0.1% versus placebo as prophylaxis for anti‐epidermal growth factor receptor‐induced acne‐like rash: a randomized left‐right comparative evaluation (appearance). Oncol. 2019;24:885–e413. 10.1634/theoncologist.2019-0156 PMC6656472 30890624

[32] Scope A , Agero ALC , Dusza SW , Myskowski PL , Lieb JA , Saltz L , et al. Randomized double‐blind trial of prophylactic oral minocycline and topical tazarotene for cetuximab‐associated acne‐like eruption. J Clin Oncol. 2007;25:5390–5396. 18048820 10.1200/JCO.2007.12.6987

[33] Ma T , Hara M , Sougrat R , Veavatz J‐M , Verkman AS . Impaired stratum corneum hydration in mice lacking epidermal water channel aquaporin‐3. J Biol Chem. 2002;277:17147–17153. 11880378 10.1074/jbc.M200925200

[34] Ikarashi N , Kaneko M , Watanabe T , Kon R , Yoshino M , Yokoyama T , et al. Epidermal growth factor receptor tyrosine kinase inhibitor erlotinib induces dry skin via decreased in aquaporin‐3 expression. J Biomol. 2020;10:545. 10.3390/biom10040545 PMC7225942 32260143

[35] Sugiyama Y , Ota Y , Hara M , Inoue S . Osmotic stress up‐regulates aquaporin‐3 gene expression in cultured human keratinocytes. Biochim Biophys Acta. 2001;1522:82–88. 11750058 10.1016/s0167-4781(01)00320-7

[36] Varani J , Fisher GJ , Kang S , Voorhees JJ . Molecular mechanisms of intrinsic skin aging and retinoid‐induced repair and reversal. J investing Dermatol Symp Proc. 1998;3:57–60. 9732060

