# Supplementary figures and images for: Mechanism of action of adapalene for treating EGFR‐TKI‐induced skin disorder
Source: Thorac Cancer. 2024 Feb 20;15(9):722–9. doi: 10.1111/1759-7714.15249 (PMC10961223; doi:10.1111/1759-7714.15249)

A(0.1%)

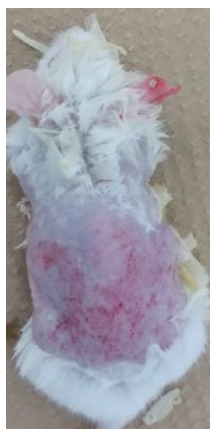

E+A(0.1%)

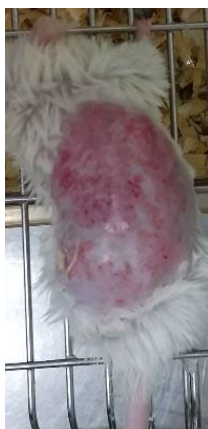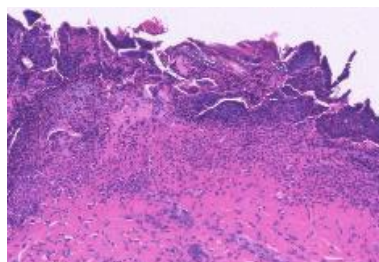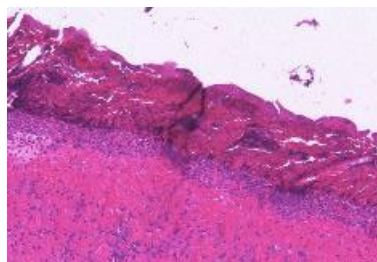

Supplement: Supplementary file 1 — FIGURE S1. Skin disorder of mice treated with 0.1% adapalene with or without afatinib. Both mice showed severe skin inflammation. [file TCA-15-722-s001.pdf]
