# Supplementary material for: Mechanism of action of adapalene for treating EGFR‐TKI‐induced skin disorder
Source: Thorac Cancer. 2024 Feb 20;15(9):722–9. doi: 10.1111/1759-7714.15249 (PMC10961223; doi:10.1111/1759-7714.15249)
Supplement: Supplementary file 2 — FIGURE S2. mRNA levels of indicated cytokines normalized to GAPDH. [file TCA-15-722-s002.pdf]

	E	E+A250
CCL2	2.5	0.8
CCL3	1.8	2.5
CCL5	-3.7	9.7
CCL18	0.5	0.0
CCL27	-0.5	-1.1
CXCL1	0.3	-1.0
CXCL9	N/D	N/D
CXCL14	-1.0	-3.3
CX3CL1	-1.0	0.8
IL-8	4.7	4.2
IL-10	0.3	2.0

(log_2 relative folds)
